# Autologous anti-SOX2 antibody responses reflect intensity but not frequency of antigen expression in small cell lung cancer

**DOI:** 10.1186/1472-6890-14-24

**Published:** 2014-06-07

**Authors:** Sukru Atakan, Hulya Bayiz, Serpil Sak, Alper Poyraz, Burcak Vural, Azmi Serhat Yildirim, Funda Demirag, Ali Osmay Gure

**Affiliations:** 1 Department of Molecular Biology and Genetics, Bilkent University, Ankara 06800, Turkey; 2 Department of Thoracic Medicine, Atatürk Chest Diseases and Chest Surgery Education and Research Hospital, Ankara, Turkey; 3 Department of Pathology, Ankara University, Ankara, Turkey; 4 Department of Pathology, Atatürk Chest Diseases and Chest Surgery Education and Research Hospital, Ankara, Turkey; 5 Department of Genetics, Institute for Experimental Medicine, Istanbul University School of Medicine, Istanbul, Turkey

**Keywords:** Tumor immunology, Cancer stem cells, Autologous antibody responses, Tumor antigens, Lung cancer, Immunohistochemistry, Autologous antibodies, Cancer stem-cells

## Abstract

**Background:**

Anti-SOX2 antibody responses are observed in about 10 to 20% of small cell lung cancer (SCLC) patients. The aim of this study was to determine whether such responses reflect a particular pattern of SOX2 protein expression in the tumor and whether this pattern associates with clinical outcome.

**Methods:**

Paraffin embedded tumor tissues, obtained from SCLC patients who had no evidence of paraneoplastic autoimmune degeneration, were evaluated for SOX2 expression by immunohistochemistry for both intensity and extent of staining. Sera from the same patients were tested for autologous antibodies against recombinant SOX2 by enzyme-linked immunosorbent assay (ELISA). Correlates between overall survival and various clinical parameters including SOX2 staining and serology were determined.

**Results:**

SOX2 protein expression was observed in tumor tissue in 89% of patients. Seventeen patients (29%) were seropositive for SOX2 antibodies and, in contrast to SOX2 staining, the presence of antibody correlated with limited disease stage (p = 0.05). SOX2 seropositivity showed a significant association with the intensity of SOX2 staining in the tumor (p = 0.02) but not with the frequency of SOX2 expressing cells.

**Conclusion:**

Anti-SOX2 antibodies associate with better prognosis (limited stage disease) while SOX2 protein expression does not; similar to reports from some earlier studies. Our data provides an explanation for this seemingly contrasting data for the first time as SOX2 antibodies can be observed in patients whose tumors contain relatively few but strongly staining cells, thus supporting the possible presence of active immune-surveillance and immune-editing targeting SOX2 protein in this tumor type.

## Background

SRY-homology box group B1 genes (*SOX1, SOX2, SOX3*) are known to function in neural plate, gut and lung development [1,2]; and *SOX2* has a role in maintaining the pluripotent stem cell phenotype [3]. In line with these facts, SOX2 protein expression was shown to be an independent marker for worse outcome in early stage lung adenocarcinoma [4] and to associate with tumor aggression and higher grade in lung cancer [5]. Another study, however, correlated SOX2 expression with lower grade and with better outcome in squamous cell carcinoma of the lung [6], and a recent study found a relation between SOX2 expression and advanced disease, as well as worse overall survival in SCLC [7]. These seemingly conflicting results could be due to tumor type specific behavior of *SOX2*, or technical reasons but they could also be due to presence of unacknowledged confounding prognostic factors. We hypothesized that such a factor could be the presence of an autologous immune response against SOX2. Cancer patients can mount antibody responses against a wide range of tumor antigens [8]. SOX Group B1 proteins have been shown to elicit some of the highest titered autologous anti-tumor antibody responses observed to date [9]. Despite the high-titered responses, several studies showed no association between immune responses to SOX proteins and improved outcome [10,11], although others did [12,13]. In this line, how anti-SOX2 antibody responses relate to SOX2 protein expression in lung cancer remains unanswered. Since anti-SOX2 antibodies are most frequently found in patients with small cell carcinoma of the lung (SCLC), we asked if immunity against SOX2 was related to its protein expression, and if either related to clinical parameters determining outcome in SCLC.

## Methods

### Patient and control population

The study cohort consisted of 59 patients with pathologically confirmed SCLC diagnosed between October 2007 and January 2009. All patients gave informed consent and the study was approved by the ethical board of the Atatürk Chest Diseases and Chest Surgery Education and Research Hospital, Ankara, Turkey. All samples were anonymized before analysis. None of the patients had neurological symptoms or evidence of paraneoplastic disease (PND) within the follow-up period. The clinical data obtained from patients at the time of diagnosis included age, gender, tumor stage, serum alkaline phosphatase (AP), and lactate dehydrogenase levels (LDH). All patients received chemotherapy with or without concurrent and sequential radiotherapy. Survival data was available for all patients. Follow-up times ranged from 0.2 to 44.7 months with a median of 8.68 months. Sera from 157 age-matched healthy controls were obtained after informed consent at the Capa Chest Diseases and Chest Surgery Education and Research Hospital of İstanbul, Turkey.

### Enzyme-linked immunosorbent assay

Serum samples were obtained from all 59 patients, collected at the time of diagnosis, between October 2007 and January 2009 and stored at −70°C. SOX2 and two control proteins (EBV-p18 and DHFR) were expressed and purified using the prokaryotic pQE expression system (Qiagen Inc., Valencia, CA, USA). All constructs contained cDNA corresponding to the full ORF of each gene. Ninety-six well Immulon 4 HBX plates (Thermo Scientific, Lafayette, CO, USA) were coated with 0.2 μg/ml antigen at 4°C for 16 hours. Plates were blocked with 5 % non-fat milk in PBS. Patient sera were added to plates at two dilutions (1:400, 1:1600) and incubated for 2 h at 37°C and subsequently with goat anti-human IgG - AP conjugate (Jackson Immunoresearch Laboratories Inc., West Grove, PA, USA) at a dilution of 1:5000. The color reaction was read with an ELISA plate reader (BioTek Instruments, Inc., Winooski, VT, USA). Each experiment was repeated twice. A sample was considered seropositive if the average of OD405 values for the two dilutions for a given sample was above the mean plus 2 standard deviation of that obtained for healthy control serum (corresponding to an OD405 value of 1,799). Six control sera (3.8%) were seropositive (Additional file 1: Figure S1). DHFR was used as negative and EBV p18 as positive controls. OD values obtained for anti-p18 antibodies from patients and controls were not statistically different.

### Western analysis of SOX2 seroreactivity

One hundred nanograms per well of recombinant SOX2 protein was separated by 12% SDS-PAGE under denaturing conditions and transferred to Immobilon-P PVDF membranes (Milipore, St. Charles, MO, USA) using the BioRad semi-dry transfer system. The membranes were then blocked in 5% non-fat milk and incubated with patient serum diluted at 1:3000 for 16 hours at 4°C, after which they were washed in TBS-T and incubated with goat anti-human IgG (Fc-specific)-HRP (Sigma, St. Louis, MO, USA ) for 2 hours. Immunoreactive protein was visualized using ECL-Plus Western Blotting system (GE Healthcare, Buckinghamshire, UK). Mouse anti-human SOX2 monoclonal antibody (R&D Systems, Minneapolis, MN, USA) was used as a positive control.

### Immunohistochemistry

Formalin-fixed paraffin embedded tumor tissues from all patients, obtained at the time of diagnosis, were retrospectively evaluated by IHC. Fifty five of 59 tissues had sufficient tumor tissue for a reliable evaluation. Tissues were sectioned at 4 μm, placed on positively charged slides and stained using a Ventana Benchmark LT automatic immunostainer (Ventana Medical Systems, Tuscon, AZ, USA). A range of dilutions of the primary antibody as well as various incubation times and temperatures were tested for optimization. Monoclonal mouse anti-human SOX2 primary antibody (MAB2018, R&D Systems, Minneapolis, MN, USA) was used to stain sections at a 1:25 dilution for 40 minutes. The iViewT DAB Detection Kit (Ventana Medical Systems, Tuscon, AZ, USA), with standard CC1 pretreatment was used for detection. IHC staining was estimated by microscopy as the frequency (percentage) of stained cells as well as by the intensity of staining (graded from 0 to 3). H-scores were calculated as described previously [14]. All samples were evaluated independently by two pathologists (SS and FD). The NTERA-2 cell line was used as a positive control and for optimization experiments. A normal tissue (sausage) block was used as a negative control and was included in every run.

### Statistical analysis

Distribution of anti-SOX2 antibody or SOX2 staining in each of age, sex, stage, AP and LDH categories were examined using frequency tables, and differences were evaluated with two sided chi-square tests. Overall survival was defined as the time from diagnosis to death or date of last follow-up. Data for patients that were alive at the last contact were censored. SOX2 protein or antibody effects on survival were estimated by Kaplan-Meier method and the log-rank test was used to compare survival across groups. All data were dichotomized as indicated in the tables and analyzed as categorical variables. All *P* values were two-sided. All analyses were performed using GraphPad Prism version 6.00, (GraphPad Software, San Diego California USA), or the Statistical Package for the Social Sciences, version 19 (SPSS Inc., Chicago, IL).

## Results

**Figure 1:**
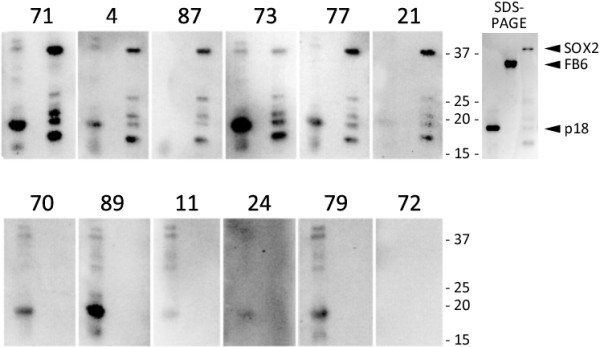
**SOX2 Western results confirm ELISA data.** Sera from patients with anti-SOX2 antibodies according to ELISA (#71, 4, 87, 73, 77, 21) and those without antibodies (#70, 89, 11, 24, 79, 17) were analyzed by Western blotting for reactivity against EBV p18 protein (positive control, 18kD), FB6 (a negative control, 35kD), and SOX2 (38kD), run in the first, second and third lanes of each gel, respectively. Coomassie blue staining of SDS-PAGE analysis of the proteins used for Western is shown on the upper right. Smaller molecular weight bands observed for SOX2 are due to premature translation termination.

**Figure 2:**
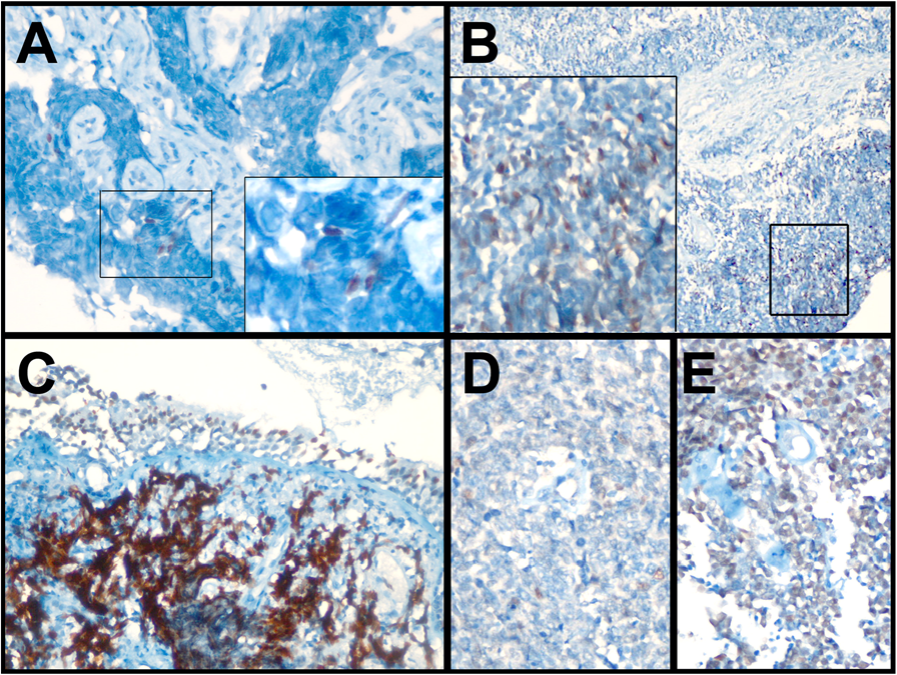
**SOX2 staining is heterogeneous.** Tumors from three seropositive (**A**-**C**; AGH-KHA-4, 71, and 21 respectively) and two seronegative patients (**D** &**E**; AGH-KHA-72, and 89, respectively) are shown. SOX2 staining is predominantly nuclear. In addition to tumor cells, staining of bronchiolar epithelial cells was also observed **(C)**. See Table 2 for details of immunohistochemical evaluation and SOX2 seropositivity testing results.

The clinical features of the 59 SCLC patients and their association with overall survival are shown in Table 1. Median age was 64 years (range, 44 to 85 years). All except 6 patients were male. Cut-off values for AP and LDH were 70 IU and 200 IU, respectively [15,16]. Fifty one percent of the patients had limited stage disease at the time of diagnosis. Limited disease stage was associated with longer overall survival (p = 0.03). Seventeen of 59 patients (29%) had antibodies against SOX2 (Table 2), as determined by ELISA using recombinant SOX2 protein, and confirmed by Western analysis (Figure 1 and Additional file 1: Figure S1). We did not observe an association of antibodies with overall survival (Additional file 1: Figure S2). However, SOX2 antibodies were more often present in serum from patients with limited stage disease: while 12 of 28 patients with limited stage had SOX2 antibodies, only 5 patients with extensive disease were seropositive (p = 0.05) (Table 3). We could not find a statistically significant correlation between SOX2 seropositivity and any other clinical parameter. Positive staining by immunohistochemistry for SOX2 protein was observed in 42 of 55 tumors and was primarily nuclear and occasionally cytoplasmic in character, ranging from very intense to weak, with frequencies between 2% to 90% (Figure 2 and Table 2). Although in most cases only some cells expressed SOX2, the intensity of staining for those cells within a given tumor was always of similar intensity. We found no statistically significant correlations between frequency or intensity of SOX2 protein expression and any of the clinical features. We then asked whether SOX2 antibodies correlated with SOX2 protein expression in tumor tissues. We found no statistically significant association between the frequency of SOX2 staining and SOX2 antibody presence, when tumors were classified based on whether they contained positively staining cells below and above a cut off of 5, 20 or 40% of the total tissue (Table 1). When evaluated for intensity of staining, all 13 patients with no SOX2 expression in their tumors were found to be seronegative for SOX2 antibodies. However, while only 2 of 12 patients with weak ("1") staining had antibodies against SOX2, 14 of the 30 patients whose tumors contained intensely staining cells ("2-3") were seropositive for anti-SOX2 (p = 0.017); suggesting that strong SOX2 expression, even if focal, might suffice in inducing an immune response against this antigen (Table 4). The mean H-score for SOX2 seropositive tumors was larger (156.8) than that for seronegatives (110.6), however, the difference was not statistically significant (p = 0.25: two sided t-test).

**Table 1:** Clinical features and SOX2 antibody and protein staining characteristics of SCLC patients

		**n**	**Median survival (months)**	**P (log rank)**
Age	61 (44–78)			
	<60	24	10.36	0.76
	≥60	35	7.1	
LDH	193 IU/L (133–380)			
	<200	30	10.75	0.36
	≥200	25	8.86	
	U*	4		
AP	93 IU/L (50-171			
	<70	14	3.97	0.47
	≥70	44	8.68	
	U	1		
Stage	Limited	28	10.36	**0.03**
	Extensive	27	7.34	
	U	1		
SOX2 antibody	<M + 2SD	42	8.68	0.3
	≥ M + 2SD	17	7.85	
SOX2 IHC intensity**	0-1	25	7.39	0.98
	2-3	30	10.65	
SOX2 IHC frequency	<5 %	16	25.87	0.12
	≥5 %	39	7.39	
SOX2 IHC frequency	<20 %	24	7.4	0.11
	≥20 %	31	7.86	
SOX2 IHC frequency	<40 %	29	7.39	0.43
	≥40 %	26	9.68	

*U: unknown. **Tumor tissue sections evaluations were available from 55 patients.

**Table 2:** SOX2 antibody and protein staining characteristics of SCLC patients

**Patients**	**SOX2 Ab (OD405)**	**SOX2 staining frequency (%)**	**SOX2 staining intensity**	**H-score**
**AGH-KHA-04**	4	2	2	6
**AGH-KHA-56**	3.93	2	3	8
**AGH-KHA-46**	3.87	UE*	UE	UE
**AGH-KHA-38**	3.84	95	3	380
**AGH-KHA-74**	3.78	100	3	400
**AGH-KHA-71**	3.77	5	2	15
**AGH-KHA-48**	3.66	30	2	90
**AGH-KHA-62**	3.62	45	2	135
**AGH-KHA-73**	3.06	10	3	40
**AGH-KHA-87**	2.93	5	2	15
**AGH-KHA-59**	2.83	90	3	360
**AGH-KHA-77**	2.74	30	2	90
**AGH-KHA-96**	2.7	60	1	120
**AGH-KHA-39**	2.42	90	3	360
**AGH-KHA-37**	1.98	10	1	20
**AGH-KHA-21**	1.97	50	2	150
**AGH-KHA-32**	1.93	80	3	320
AGH-KHA-93	1.78	2	1	4
AGH-KHA-76	1.68	0	0	0
AGH-KHA-81	1.28	5	1	10
AGH-KHA-28	1.23	0	0	0
AGH-KHA-90	1.2	80	2	240
AGH-KHA-17	1.2	0	0	0
AGH-KHA-66	1.19	0	0	0
AGH-KHA-75	1.18	25	1	50
AGH-KHA-80	1.15	UE	UE	UE
AGH-KHA-11	1.09	20	2	60
AGH-KHA-29	1.09	50	2	150
AGH-KHA-86	1.08	5	1	10
AGH-KHA-95	1.07	50	1	100
AGH-KHA-89	1.04	90	2	270
AGH-KHA-78	1.03	0	0	0
AGH-KHA-70	1.03	20	1	40
AGH-KHA-67	1.01	80	2	240
AGH-KHA-45	0.95	0	0	0
AGH-KHA-68	0.92	95	3	380
AGH-KHA-69	0.87	5	1	10
AGH-KHA-91	0.85	40	2	120
AGH-KHA-88	0.84	0	0	0
AGH-KHA-94	0.81	10	1	20
AGH-KHA-33	0.79	0	0	0
AGH-KHA-79	0.76	90	3	360
AGH-KHA-61	0.76	90	3	360
AGH-KHA-82	0.72	0	0	0
AGH-KHA-57	0.68	UE	UE	UE
AGH-KHA-53	0.65	70	2	210
AGH-KHA-55	0.65	70	2	210
AGH-KHA-15	0.64	0	0	0
AGH-KHA-40	0.64	60	2	180
AGH-KHA-64	0.63	70	2	210
AGH-KHA-72	0.61	40	1	80
AGH-KHA-24	0.55	90	3	360
AGH-KHA-51	0.55	60	3	240
AGH-KHA-43	0.54	0	0	0
AGH-KHA-12	0.52	UE	UE	UE
AGH-KHA-50	0.5	0	0	0
AGH-KHA-52	0.48	70	3	280
AGH-KHA-34	0.46	60	1	120
AGH-KHA-49	0.45	0	0	0

*UE: Unable to evaluate. Seropositive sera (OD405 > 1,799) are indicated in bold font.

**Table 3:** SOX2 antibody correlates with clinical stage

		**SOX2 Ab**		**P (chi.sq.)**
		**(−)**	**(+)**	
Stage	Limited	16	12	0.05
	Extensive	22	5	

**Table 4:** SOX2 antibody correlates with intensity of SOX2 protein expression

		**SOX2 Ab**		**P (chi.sq.)**
		**(−)**	**(+)**	
SOX2 IHC intensity	0-1	23	2	0.017
	2-3	16	14	

## Discussion

The illumination of the mechanisms underlying anti-tumor immune responses is critical as such responses can be ultimately boosted if their beneficial affect can be proven. To our knowledge, this is the first study where autologous anti-tumor antibody responses against SOX2 have been correlated with tumor antigen expression in SCLC. It is known that autologous antibodies can be elicited against either upregulated, mutated or foreign proteins [17]. SOX2 is amplified and thus upregulated, but not mutated in SCLC [18]. In patients with monoclonal gammopathy of undetermined significance (MGUS), anti-SOX2 T cell responses were found to be directed against a very small percentage of tumor cells which were of the clonogenic type [13]. In the same study, patients with anti-SOX2 T cells showed significantly better overall survival. In contrast to the case with MGUS, in SCLC, SOX2 immune responses, as measured by the presence of antibody, have been observed to correlate with better outcome only in one of the three cohorts studied so far [10-12]. We believe there could be several explanations for this. Firstly, as demonstrated in the MGUS study, anti-SOX2 T-cell and antibody responses do not overlap completely; therefore, if T-cell responses determine outcome more so than antibodies, than this awaits to be demonstrated for SCLC. Secondly, no study to date has shown an association between worse outcome, or prognosis with SOX2 seropositivity. In contrast, SOX2 protein expression has been related to more aggressive tumors in several studies [4,19-21] and the upregulation of this gene is known to enhance tumor cell proliferation [18]. In addition, *SOX2* overexpression has been shown to be essential for lung cancer stem cell function [22,23]. It is possible that antibody responses are associated with improved outcome, but given the fact that SOX2 expression has the opposite effect, the two cancel out each other. Larger and comprehensive studies might further clarify these matters.

A very strong evidence that anti-tumor immune responses can effectively eliminate selected cells in humans comes from the observation that anti-tumor responses against antigens expressed in tumors but also in normal tissues, and in particular in Purkinje cells of the cerebellum, are able to completely eliminate these cells, without destroying surrounding tissues [24]. The amount of antigen expressed by a given cell has been shown to determine the type of T cell response directed against it [25]. HER-2/neu specific immunity, for example, has been shown to depend on the antigens' expression level [26]. Therefore, it is possible that only cells with intense SOX2 expression were able to induce immune responses against them in the cohort we studied, potentially resulting in their loss, similar to the case with Purkinje cells in patients with anti-HuD antibodies. The fact that in our study SOX2 seropositive patients' tumors on occasion contained very few cells could be reflecting the fact that such tumors undergo immune-editing [27], and reach a state of equilibrium between the tumor and the immune response following a loss of most SOX2 expressing cells [28]. Another explanation could be that those cells in tumors from patients who are seropositive represent clonogenic cells which elicit an immune response as observed for MGUS patients [13]. In glioma the intensity and not the frequency of SOX2 expression was shown to be an indicator of a more stem-like phenotype [29]. On the other hand, the weak but diffuse staining in a number of tumors we studied might reflect the presence of SOX2 expressing non-clonogenic cells. This is likely as we and others have found morphologically normal bronchial epithelium to be frequently positive for SOX2 [30], and is also supported by experiments showing that the transfection of a second gene in addition to *SOX2* is required for tumorigenic transformation in some models [31,32].

## Conclusions

We report, for the first time, a relation between SOX2 protein expression characteristics and anti-SOX2 antibody responses in patients with SCLC. Although we find no correlation between outcome or clinical measures with frequency or intensity of SOX2 expression, we observe that SOX2 seropositivity associates with better prognosis. Tumors from patients with SOX2 antibody generally contain strongly staining cells, in contrast to tumors from seronegative patients. This suggests that the intensity of SOX2 expression might be critical in eliciting an anti-SOX2 immune response. The fact that several tumors have very low numbers of such cells suggests that SOX2 expressing cells could have been eliminated over the course of disease, which is in support of active tumor immune-surveillance and immune-editing in these patients.

## Competing interests

The authors declare that they have no competing interests. AOG holds a patent on the use of SOX2 and related molecules (US 7314721).

## Authors’ contributions

Conception and design: SA, HB, FD, BV, AOG. Data acquisition: SA, HB, SS, FD, ASY. Data analysis/interpretation: SA, AP, HB, SS, FD, AOG. Drafting and final approval: SA, HB, SS, AP, BV, FD, ASY, AOG. All authors read and approved the final manuscript.

## Pre-publication history

The pre-publication history for this paper can be accessed here:

http://www.biomedcentral.com/1472-6890/14/24/prepub

## Supplementary Material

Additional file 1: Figure S1 Scatter dot plot of SOX2 ELISA. The median values for small cell lung cancer (SCLC) and healthy control sera (CTR), as well as the cut-off for seropositivity (dotted line) are shown. **Figure S2.** Kaplan-Meier analysis of patients stratified according to SOX2 seropositivity. Although seropositive patients show a trend for better overall survival the difference, as calculated by the log-rank test, is insignificant (p=0.3). Click here for file
