# Supplementary material for: Autologous anti-SOX2 antibody responses reflect intensity but not frequency of antigen expression in small cell lung cancer
Source: BMC Clin Pathol. 2014 Jun 7;14:24. doi: 10.1186/1472-6890-14-24 (PMC4060123; doi:10.1186/1472-6890-14-24)
Supplement: Additional file 1: Figure S1 — Scatter dot plot of SOX2 ELISA. The median values for small cell lung cancer (SCLC) and healthy control sera (CTR), as well as the cut-off for seropositivity (dotted line) are shown. Figure S2. Kaplan-Meier analysis of patients stratified according to SOX2 seropositivity. Although seropositive patients show a trend for better overall survival the difference, as calculated by the log-rank test, is insignificant (p=0.3). [file 1472-6890-14-24-S1.docx]

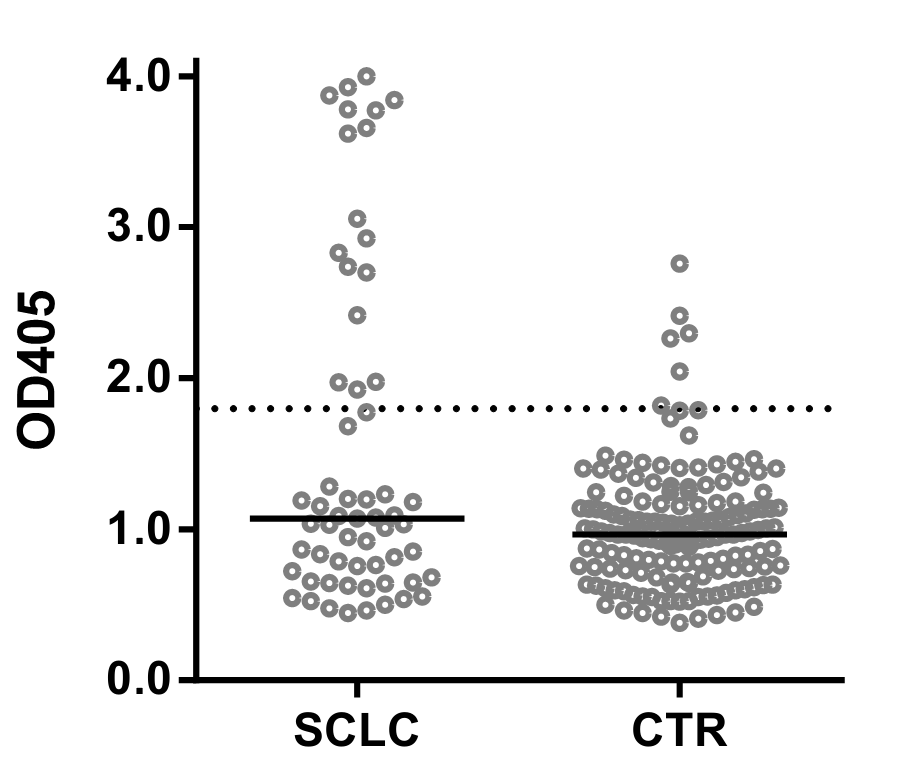


**Supplementary Figure 1. Scatter dot plot of SOX2 ELISA.** The median values for small cell lung cancer (SCLC) and healthy control sera (CTR), as well as the cut-off for seropositivity (dotted line) are shown.


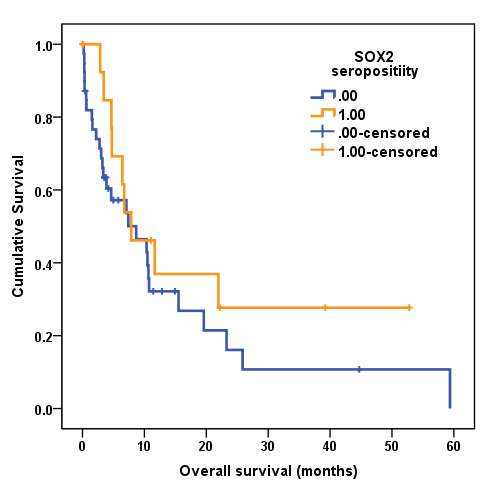


**Supplementary Figure 2. Kaplan-Meier analysis of patients stratified according to SOX2 seropositivity.** Although seropositive patients show a trend for better overall survival the difference, as calculated by the log-rank test, is insignificant (p=0.3).
